# Machine Learning for the Prediction of Complications in Patients After Mitral Valve Surgery

**DOI:** 10.3389/fcvm.2021.771246

**Published:** 2021-12-16

**Authors:** Haiye Jiang, Leping Liu, Yongjun Wang, Hongwen Ji, Xianjun Ma, Jingyi Wu, Yuanshuai Huang, Xinhua Wang, Rong Gui, Qinyu Zhao, Bingyu Chen

**Affiliations:** ^1^Clinical Laboratory, The Third Xiangya Hospital, Central South University, Changsha, China; ^2^Hunan Engineering Technology Research Center of Optoelectronic Health Detection, Changsha, China; ^3^Department of Transfusion, The Third Xiangya Hospital, Central South University, Changsha, China; ^4^Department of Blood Transfusion, The Second Xiangya Hospital, Central South University, Changsha, China; ^5^Department of Anesthesiology, Fuwai Hospital National Center for Cardiovascular Diseases, Chinese Academy of Medical Sciences, Peking Union Medical College, Beijing, China; ^6^Department of Blood Transfusion, Qilu Hospital of Shandong University, Jinan, China; ^7^Department of Transfusion, Xiamen Cardiovascular Hospital Xiamen University, Xiamen, China; ^8^Department of Transfusion, The Affiliated Hospital of Southwest Medical University, Luzhou, China; ^9^Department of Transfusion, Beijing Aerospace General Hospital, Beijing, China; ^10^College of Engineering & Computer Science, Australian National University, Canberra, ACT, Australia; ^11^Department of Transfusion, Zhejiang Provincial People's Hospital, Hangzhou, China

**Keywords:** machine learning, cardiac valvular surgery, complications, predict, model

## Abstract

**Background:** This study intended to use a machine learning model to identify critical preoperative and intraoperative variables and predict the risk of several severe complications (myocardial infarction, stroke, renal failure, and hospital mortality) after cardiac valvular surgery.

**Study Design and Methods:** A total of 1,488 patients undergoing cardiac valvular surgery in eight large tertiary hospitals in China were examined. Fifty-four perioperative variables, such as essential demographic characteristics, concomitant disease, preoperative laboratory indicators, operation type, and intraoperative information, were collected. Machine learning models were developed and validated by 10-fold cross-validation. In each fold, Recursive Feature Elimination was used to select key variables. Ten machine learning models and logistic regression were developed. The area under the receiver operating characteristic (AUROC), accuracy (ACC), Youden index, sensitivity, specificity, F1-score, positive predictive value (PPV), and negative predictive value (NPV) were used to compare the prediction performance of different models. The SHapley Additive ex Planations package was applied to interpret the best machine learning model. Finally, a model was trained on the whole dataset with the merged key variables, and a web tool was created for clinicians to use.

**Results:** In this study, 14 vital variables, namely, intraoperative total input, intraoperative blood loss, intraoperative colloid bolus, Classification of New York Heart Association (NYHA) heart function, preoperative hemoglobin (Hb), preoperative platelet (PLT), age, preoperative fibrinogen (FIB), intraoperative minimum red blood cell volume (Hct), body mass index (BMI), creatinine, preoperative Hct, intraoperative minimum Hb, and intraoperative autologous blood, were finally selected. The eXtreme Gradient Boosting algorithms (XGBOOST) algorithm model presented a significantly better predictive performance (AUROC: 0.90) than the other models (ACC: 81%, Youden index: 70%, sensitivity: 89%, specificity: 81%, F1-score:0.26, PPV: 15%, and NPV: 99%).

**Conclusion:** A model for predicting several severe complications after cardiac valvular surgery was successfully developed using a machine learning algorithm based on 14 perioperative variables, which could guide clinical physicians to take appropriate preventive measures and diminish the complications for patients at high risk.

## Introduction

The prevalence of unhealthy lifestyles, such as long-term high-fat diet and lack of exercise, has caused the higher and higher incidence of cardiac diseases. Patients with cardiac diseases will suffer serious morbidity and mortality without reasonable interventions, which increased the number of cardiac surgery significantly. It was discovered that more than 1 million patients with heart disease need to be treated with cardiac surgery every year worldwide (1). There has also been a sharp increase in the number of patients with valvular diseases, many of which are severe and must be treated with cardiac surgery to replace insufficient valves (2, 3). A large number of cardiac patients always along with various of complications after cardiac valvular surgery, these postoperative complications mainly including myocardial infarction, stroke, acute renal failure, death, and so on (4).

The high incidence of postoperative complications in cardiac surgery plays an important role in the exacerbation of hospital stay and hospitalization cost, reducing the quality of life and even elevating mortality after cardiac surgery (5). An eligible surgical treatment involves not only a smooth operation but also the early prediction of risks, provision of appropriate recommendations, and timely adoption of effective medical measures to avoid postoperative complications (6).

The most important process for a qualified medical treatment is the early prediction of postoperative complications (7). Clinicians generally give judgments whether patients have postoperative complications mainly based on the tests of clinical laboratory and examinations, or their clinical experiences, when patients have corresponding clinical indications after cardiac surgery (8). On the one hand, the tests or examinations for postoperative complications are time-sensitive; on the other hand, clinical experience is subjective, and many young clinicians do not have mature clinical experience. Based on the above situation, patients who underwent cardiac surgery always miss the optimal treatment window for postoperative complications. Thus, it is urgent to construct a risk predictive system that could implement the best outcome for patients.

Previous studies on predicting postoperative risks after cardiac surgery mainly on account of traditional stastics methods, such as linear models or logistic regression (9). However, these traditional methods usually focus on one or few clinical indicators. More and more studies have found that the preoperative and intraoperative indicators of a patient have an impact on the outcome of the patient (10). Meanwhile, many studies have proved that a prediction model based on machine learning has high accuracy in predicting clinical outcomes (11, 12). Therefore, we aim to construct a model based on machine learning to predict the postoperative outcomes of patients using various preoperative and intraoperative indicators, so as to provide theoretical guidance for clinical practice.

The purpose of this study was to determine the preoperative and intraoperative risk factors associated with postoperative complications in patients undergoing cardiac valvular surgery and to develop a machine learning model to predict postoperative complications.

## Materials and Methods

### Study Subjects

Participants were patients aged more than 18 years but <75 who underwent cardiac valvular surgery (mitral valve replacement, mitral valvuloplasty, and tricuspid valvuloplasty) from January 2016 to December 2018 at one of the following eight tertiary hospitals: the Second Xiangya Hospital of Central South University, the Third Xiangya Hospital of Central South University, Beijing Aerospace General Hospital, Qilu Hospital of Shandong University, Fuwai Hospital National Center for Cardiovascular Diseases, Zhejiang Provincial People's Hospital, the Affiliated Hospital of Southwest Medical University, and Xiamen Cardiovascular Hospital Xiamen University. We collected 38 cases of biological valve replacement from the Third Xiangya Hospital from 2019 to 2020 for verification.

The types of surgery for cardiac valvular surgery in our study include the classical mitral valve replacement, mitral valvuloplasty, and tricuspid valvuloplasty. Since the four types of surgery account for the majority of the population, only these three procedures were included in this study.

Patients who underwent other types of surgery (coronary artery bypass grafting, CABG, atrial septal defect repair, etc.), re-cardiac surgery, or emergency surgery, and those whose missing rates of data were more than 80% were excluded.

Postoperative myocardial infarction, postoperative stroke, postoperative renal failure, and postoperative hospital mortality that occurred 48 h after the initial surgery were defined as relevant outcomes; Then we labeled patients who had at least one complication as “complication occurred” and patients who did not have any complication as “complication did not occur.”

Approval was obtained from the institutional review board of the Third Xiangya Hospital of Central South University for this study (NCT03885570). The study was reported according to the recommendations of the Transparent Reporting of a multivariable prediction model for Individual Prognosis Or Diagnosis (TRIPOD) statement. No written consent was required in view of the purely observational nature of the study.

### Study Design and Data Collection

A total of 54 preoperative variables (within 24 h before the day of surgery), intraoperative variables, and postoperative variables (occurred 48 h after the initial surgery) were collected. For some preoperative variables with multiple measurements, the values closest to the start time of the surgery were assessed. The collected preoperative information included the demographic characteristics of the patients (gender, age, and body mass index, BMI), clinical characteristics (blood group, atrial fibrillation, LV dilatation), concomitant disease (hypertension, diabetes, anemia, cerebrovascular disease), history of drug use (drug for anemia), preoperative laboratory indicators (red blood cell, RBC, white blood cell WBC), hemoglobin (Hb), red blood cell volume (Hct), platelet (PLT), creatinine, total protein (TP), albumin, globulin, alanine aminotransferase (ALT), aspartate aminotransferase (AST), prothrombin time (PT), international normalized ratio (INR), fibrinogen (FIB), left ventricular ejection fractions (LVEF), preoperative transfusion of RBC, preoperative transfusion of fresh frozen plasma (FFP), preoperative transfusion of PLT, preoperative transfusion of cryoprecipitate, operation type (mitral valve replacement, mitral valvuloplasty, and tricuspid valvuloplasty), intraoperative information (operation time; cardiopulmonary bypass precharge CPB, time; aortic cross clamp time; cardiopulmonary bypass precharge; blood loss; crystal infusion volume; colloid bolus; urine output; total output; total input; autologous blood; machine blood; minimum oxygen saturation, SaO_2_; minimum Hb; minimum Hct; intraoperative transfusion of RBC; intraoperative transfusion of FFP; intraoperative transfusion of PLT; intraoperative transfusion of cryoprecipitate), and others (Classification of New York Heart Association, NYHA, heart function; The American Society of Anesthesiologists, ASA, classification). All the variables were obtained from the electronic health record systems of the eight hospitals. Two authors (LL and HJ) had access to the systems and collected the data.

The data collected by different hospitals were converted and unified. For example, 1 mg/dl of creatinine is equal to 88.4 μmol/l. The three main types of operation were transformed into ordinal variables: mitral valve replacement, mitral valvuloplasty, and tricuspid valvuloplasty.

### Statistical Analysis

Continuous variables between complication and non-complication groups were compared by either the Student *t*-test or the rank-sum test as appropriate. The chi-square test or Fisher's exact test was performed to compare the differences in the categorical variables.

Then, the recursive feature elimination (RFE) algorithm was used to identify crucial variables, and we developed a machine learning model named eXtreme Gradient Boosting (XGBOOST) (13–15). In brief, RFE is a feature selection way that recursively fits a model derived from smaller feature sets until a specified termination criterion is reached. In each loop, features are graded by their importance in the trained model. By recursively eliminating one feature with the lowest importance, RFE intends to eliminate dependencies and collinearity that maybe existing in the model. Lastly, the most important features were screened out, and the XGBOOST model was developed based on the feature set. Other features were not included, because they only brought a small increment in AUROC but significantly increased the difficulty of model applications. The proposed prediction model was built in the XGBoost package in Python language, and it was carried out using the 10-fold cross-validation method, and then the AUROC was calculated.

Besides, 10 other models, CatBoost, LightGBM, MLP, SVM, LR, Random Forest, Gradiant Boosting, KNN, AdaBoost, and Naive Bayes, were developed and compared with the proposed machine learning model. These models were also developed and validated by 10-fold cross-validation, and then the AUCs were calculated. The accuracy (ACC), Youden index, sensitivity, specificity F1 score, positive predictive value (PPV), and negative predictive value (NPV) were also analyzed.

Finally, the key variables identified by REF in each fold were merged, and the 15 most important variables were selected. The XGBOOST model was trained on the whole dataset using the merged variables. After the model was established, the SHapley Additive exPlanations (SHAP) package in Python was used to explain the model by analyzing two cases. The SHAP package interpreted the output of the machine learning model using a game-theoretic approach (16). For each prediction sample, the model connected optimal credit allocation with local explanations. Besides, a web tool was created for clinicians to use our model.

## Results

### Study Population

**Figure 1:**
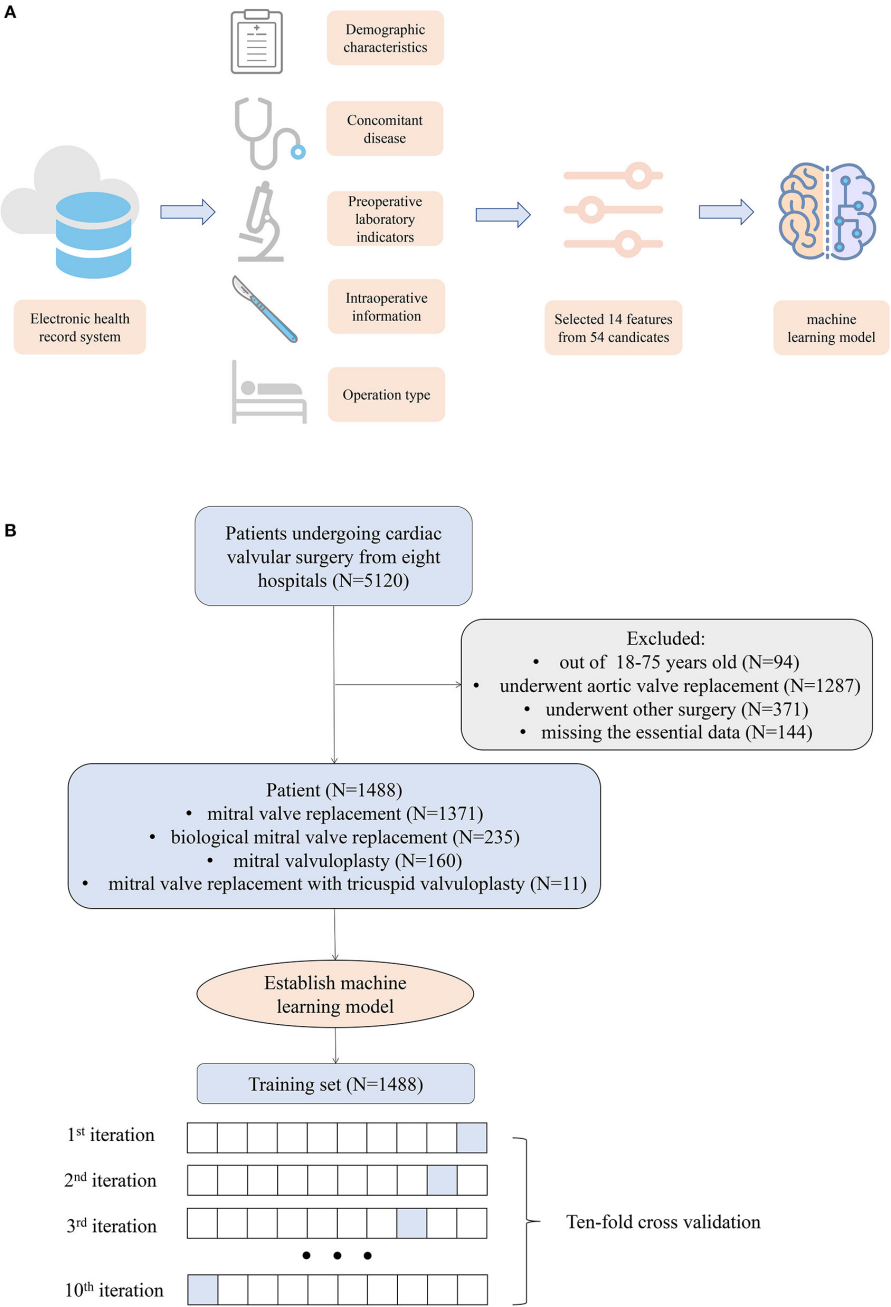
**(A)** Workflow of the study. **(B)** Flow chart of patient selection.

As Figure 1 demonstrates, 1,488 patients were finally included in this study, and the preoperative information of the cohort is described in Table 1. The average age of the patients was 52.59 years, men accounted for 39.05%, and the average BMI was 22.84. In the complication occurred cohort, 12.73% of the patients died in the hospital, 61.82% of the patients had a myocardial infarction after the operation, 30.91% of the patients had a stroke, and 74.55% of the patients had renal failure after the operation.

**Table 1:** Preoperation and intraoperative information.

**Variable**		**All (*n* = 1,488)**	**None complication group (*n* = 1,433)**	**Complication group (*n* = 55)**	***p*-value**
*n*		1,488	1,433	55	
Gender, *n* (%)	Female	907 (60.95)	873 (60.92)	34 (61.82)	0.994
	Male	581 (39.05)	560 (39.08)	21 (38.18)	
Age, mean (SD)		52.69 (10.36)	52.44 (10.24)	59.00 (11.44)	<0.001
BMI, mean (SD)		22.84 (3.39)	22.85 (3.39)	22.55 (3.45)	0.541
Blood group, *n* (%)	A	494 (33.20)	474 (33.08)	20 (36.36)	0.436
	AB	125 (8.40)	123 (8.58)	2 (3.64)	
	B	350 (23.52)	334 (23.31)	16 (29.09)	
	O	519 (34.88)	502 (35.03)	17 (30.91)	
Atrial fibrillation, *n* (%)	1	765 (51.41)	734 (51.22)	31 (56.36)	0.541
LV dilatation, *n* (%)	1	653 (43.88)	633 (44.17)	20 (36.36)	0.314
Hypertension, *n* (%)	0	1,259 (84.61)	1,217 (84.93)	42 (76.36)	<0.001
	1	100 (6.72)	88 (6.14)	12 (21.82)	<0.001
	2	60 (4.03)	59 (4.12)	1 (1.82)	<0.001
	3	69 (4.64)	69 (4.82)	0 (0.00)	<0.001
Diabetes, *n* (%)	0	1,433 (96.30)	1,378 (96.16)	55 (100.00)	0.334
	I type	14 (0.94)	14 (0.98)	0 (0.00)	0.334
	II type	41 (2.76)	41 (2.86)	0 (0.00)	0.334
Anemia, *n* (%)	1	481 (32.33)	460 (32.10)	21 (38.18)	0.424
Drug for anemia, *n* (%)	1	5 (0.34)	4 (0.28)	1 (1.82)	0.172
Cerebrovascular disease, *n* (%)	1	1,485 (99.80)	1,430 (99.79)	55 (100.00)	1
Mechanical valve, *n* (%)	1	1,082 (72.72)	1,056 (73.69)	26 (47.27)	<0.001
Mitral valvuloplasty, *n* (%)	1	160 (10.75)	155 (10.82)	5 (9.09)	0.854
Biological valve, *n* (%)	1	235 (15.79)	211 (14.72)	24 (43.64)	<0.001
NYHA, *n* (%)	1.0	24 (1.70)	23 (1.66)	1 (3.23)	<0.001
	2.0	286 (20.21)	282 (20.38)	4 (12.90)	<0.001
	3.0	971 (68.62)	955 (69.00)	16 (51.61)	<0.001
	4.0	134 (9.47)	124 (8.96)	10 (32.26)	<0.001
ASA, *n* (%)	1	22 (1.48)	5 (0.35)	17 (30.91)	<0.001
	2	75 (5.04)	62 (4.33)	13 (23.64)	<0.001
	3	1,046 (70.30)	1,030 (71.88)	16 (29.09)	<0.001
	4	345 (23.19)	336 (23.45)	9 (16.36)	<0.001
Op time (min), median [Q1,Q3]		225.00 [190.00, 265.00]	221.00 [190.00, 263.00]	291.50 [240.00, 350.00]	<0.001
CPB time (min), median [Q1,Q3]		93.00 [74.00, 118.00]	93.00 [73.75, 117.00]	117.00 [89.50, 149.50]	<0.001
Aortic cross clamp time (min), median [Q1,Q3]		59.00 [43.25, 80.00]	58.00 [43.00, 79.00]	72.00 [59.00, 95.00]	<0.001
Cardiopulmonary bypass precharge (ml), median [Q1,Q3]		1600.00 [1505.00, 1762.50]	1600.00 [1505.00, 1800.00]	1600.00 [1600.00, 1600.00]	0.103
Blood loss op (ml), median [Q1,Q3]		600.00 [420.00, 600.00]	600.00 [450.00, 600.00]	400.00 [300.00, 600.00]	<0.001
Crystal infusion volume op (ml), median [Q1,Q3]		2100.00 [1025.00, 2650.00]	2165.00 [1100.00, 2660.00]	1500.00 [1000.00, 2000.00]	0.008
Colloid bolus op (ml), median [Q1,Q3]		300.00 [0.00, 850.00]	320.00 [0.00, 1000.00]	0.00 [0.00, 0.00]	<0.001
Urine output op (ml), median [Q1,Q3]		700.00 [400.00, 1000.00]	700.00 [420.00, 1000.00]	450.00 [300.00, 752.50]	0.001
Total output op (ml), median [Q1,Q3]		2555.00 [1100.00, 3400.00]	2600.00 [1200.00, 3420.00]	0.00 [0.00, 1500.00]	<0.001
Total input op (ml), median [Q1,Q3]		2916.68 [2400.00, 3650.00]	2950.00 [2410.00, 3700.00]	2000.00 [1500.00, 2570.00]	<0.001
Autologous blood op (ml), median [Q1,Q3]		0.00 [0.00, 250.00]	0.00 [0.00, 250.00]	0.00 [0.00, 0.00]	<0.001
Machine blood, median [Q1,Q3]		800.00 [500.00, 1000.00]	800.00 [500.00, 1000.00]	500.00 [400.00, 925.00]	0.017
SO2 min op (%), median [Q1,Q3]		97.70 [94.00, 99.70]	97.50 [94.00, 99.70]	98.15 [95.95, 99.18]	0.706
RBC (10^12^/l), mean (SD)		4.50 (0.67)	4.51 (0.67)	4.32 (0.71)	0.054
WBC (10^9^/l), mean (SD)		6.61 (3.38)	6.64 (3.42)	5.82 (1.79)	0.002
HB (g/l), mean (SD)		130.18 (20.85)	130.34 (20.69)	126.16 (24.62)	0.220
HCT (/l), mean (SD)		40.37 (5.60)	40.40 (5.56)	39.54 (6.56)	0.345
Hb min op, mean (SD)		84.58 (16.63)	84.45 (16.70)	87.91 (14.57)	0.092
HCT min op, mean (SD)		24.75 (4.97)	24.66 (4.98)	27.03 (4.23)	<0.001
PLT (10^9^/l), median [Q1,Q3]		193.50 [155.00, 241.00]	194.00 [156.00, 241.25]	160.00 [116.50,234.00]	0.002
Creatinine (μmol/l), median [Q1,Q3]		71.80 [60.80, 85.00]	71.50 [60.60, 85.00]	76.90 [69.22, 92.67]	0.002
TP (g/l), median [Q1,Q3]		68.10 [63.80, 72.80]	68.10 [63.80, 72.72]	68.95 [65.82, 73.42]	0.275
Albumin (g/l), mean (SD)		39.88 (4.56)	39.92 (4.54)	38.86 (4.94)	0.126
Globulin (g/l), median [Q1,Q3]		28.00 [25.10, 31.50]	27.90 [25.00, 31.50]	29.85 [27.70, 33.35]	0.002
ALT (IU/l), median [Q1,Q3]		19.85 [13.00, 31.00]	19.90 [13.00, 31.22]	19.00 [14.00, 26.75]	0.508
AST (IU/l), median [Q1,Q3]		22.75 [18.00, 29.48]	22.70 [18.00, 29.33]	25.00 [20.00, 30.85]	0.095
PT (s), median [Q1,Q3]		13.10 [12.00,14.40]	13.20 [12.00,14.40]	11.75 [10.90,13.40]	<0.001
INR, median [Q1,Q3]		1.06 [1.00, 1.18]	1.06 [1.00, 1.18]	1.13 [1.06, 1.79]	<0.001
FIB (g/l), median [Q1,Q3]		2.90 [2.44, 3.49]	2.91 [2.44, 3.48]	2.86 [2.48, 3.71]	0.924
LVEF (%), median [Q1,Q3]		62.00 [57.00, 67.00]	62.00 [57.00, 67.00]	61.00 [56.00, 65.25]	0.152
Trans RBC before (u), median [Q1,Q3]		0.00 [0.00, 0.00]	0.00 [0.00, 0.00]	0.00 [0.00, 0.00]	0.048
Trans FFP before (ml), median [Q1,Q3]		0.00 [0.00, 0.00]	0.00 [0.00, 0.00]	0.00 [0.00,0.00]	0.603
Trans PLT before, median [Q1,Q3]		0.00 [0.00, 0.00]	0.00 [0.00, 0.00]	0.00 [0.00, 0.00]	0.001
Trans cryoprecipitate before (U), median [Q1,Q3]		0.00 [0.00, 0.00]	0.00 [0.00, 0.00]	0.00 [0.00, 0.00]	0.845
Trans RBC op (U), median [Q1,Q3]		0.00 [0.00, 0.00]	0.00 [0.00, 0.00]	0.00 [0.00, 1.75]	0.065
Trans FFP op (ml), median [Q1,Q3]		0.00 [0.00, 0.00]	0.00 [0.00, 0.00]	0.00 [0.00, 290.00]	0.010
Trans PLT op, median [Q1,Q3]		0.00 [0.00, 0.00]	0.00 [0.00, 0.00]	0.00 [0.00, 0.00]	0.628
Trans cryoprecipitate op (U), median [Q1,Q3]		0.00 [0.00, 0.00]	0.00 [0.00, 0.00]	0.00 [0.00, 0.00]	0.842

*SD, standard deviation; RBC, red blood cell; WBC, white blood cell; Hb, hemoglobin; Hct, red blood cell volume; PLT, platelet; TP, total protein, ALT, alanine aminotransferase; AST, aspartate aminotransferase; PT, prothrombin time; INR, international normalized ratio; FIB, fibrinogen; LVEF, left ventricular ejection fractions; FFP, fresh frozen plasma; CPB, cardiopulmonary bypass precharge; SaO_2_, oxygen saturation; NYHA, New York Heart Association; ASA, The American Society of Anesthesiologists; op means the intraoperative variable*.

### Key Variables

**Figure 2:**
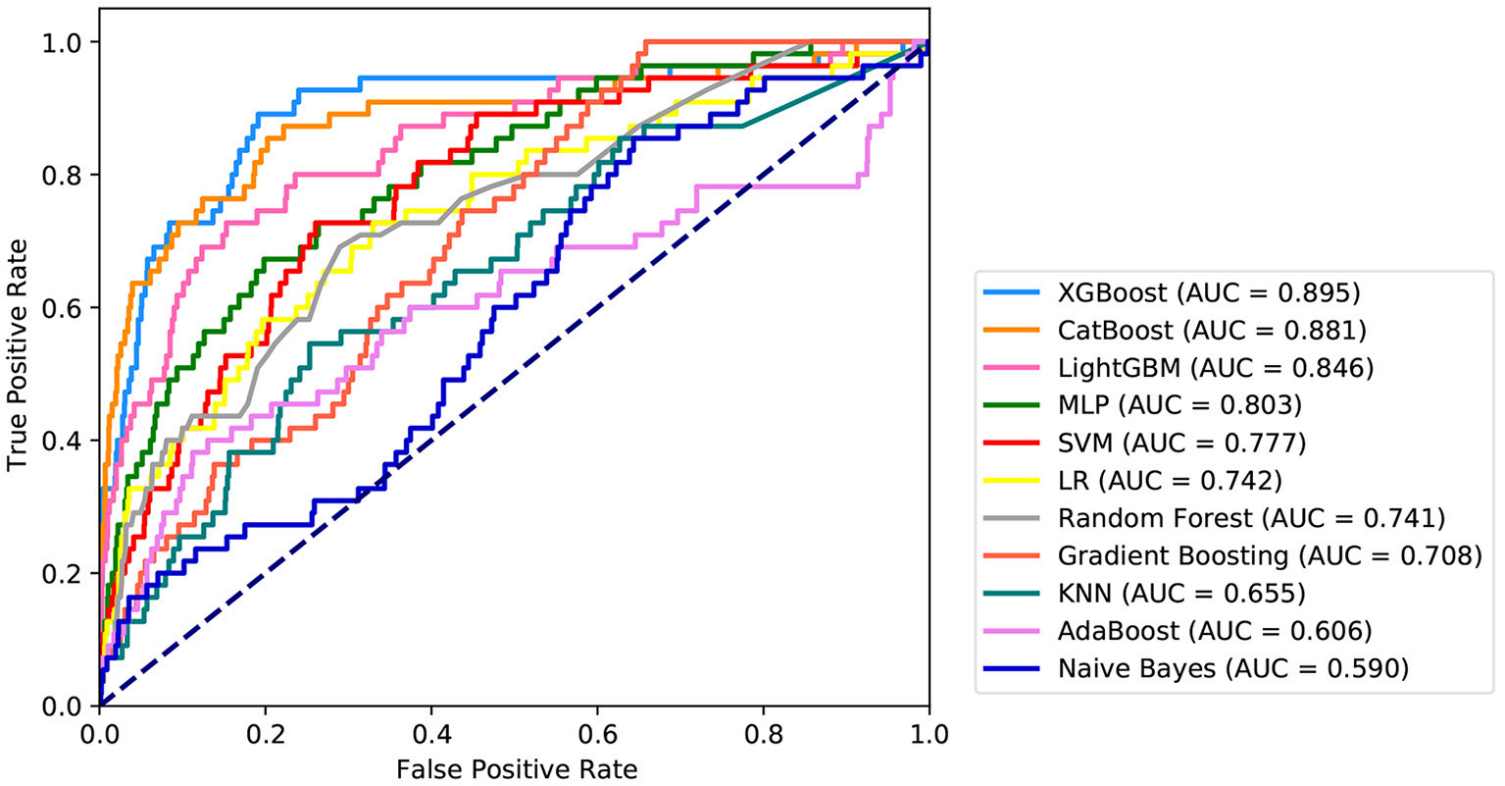
Receiver operating characteristic curves for the machine learning model and logistic regression. XGBOOST, eXtremely Gradient Boosting; CatBoost, Categorical Boosting; LightGBM, Light Gradient Boosting; MLP, Multi-Layer Perceptron; SVM, Support Vector Machine; LR, Logistic Regression; KNN, K-Nearest Neighbor; AdaBoost, Adaptive boosting.

Fifteen variables, namely, intraoperative total input, intraoperative blood loss, intraoperative colloid bolus, NYHA, preoperative Hb, preoperative PLT, age, preoperative FIB, intraoperative minimum Hct, BMI, preoperative creatinine, preoperative Hct, intraoperative minimum Hb, intraoperative, and autologous blood were selected as crucial variables using the RFE algorithm. As expected, the patients had less intraoperative total output, hypertension, higher preoperative FIB, less intraoperative total input, higher preoperative creatinine, less intraoperative autologous blood, higher NYHA score, older age, higher intraoperative minimum HCT, lower preoperative Hb, lower preoperative PLT, lower intraoperative infusion volume, higher intraoperative minimum Hb, lower preoperative HCT, higher BMI, and lower intraoperative blood loss. After identifying the 15 variables, machine learning was used to predict several severe complications after cardiac valvular surgery. As shown in Figure 2, the AUC of the proposed model is 0.9. The proposed model significantly outperformed the conventional LR (AUC: 0.74) and seven other machine learning models. As described in Table 2, ACC, Youden index, sensitivity, specificity, F1-score, PPV, and NPV of the XGBoost model is 81, 70, 89, 81, 0.26, 15, and 99%, respectively. These indicators of LR were 67, 40, 69, 71, 0.15, 8, and 98%, respectively.

**Table 2:** Performance of machine learning models.

**Model**	**AUC**	**ACC (%)**	**Youden index (%)**	**Sensitivity (%)**	**Specificity (%)**	**F1 score**	**PPV (%)**	**NPV (%)**
XGBoost	0.90	81	70	89	81	0.26	15	99
CatBoost	0.88	80	65	86	80	0.24	14	99
LightGBM	0.85	84	57	73	85	0.25	15	99
MLP	0.80	80	47	67	80	0.19	11	98
SVM	0.78	74	47	73	74	0.17	10	99
LR	0.74	67	40	73	67	0.14	8	98
Random forest	0.74	71	40	69	71	0.15	8	98
Gradient boosting	0.71	37	34	100	34	0.10	5	100
KNN	0.66	74	29	55	75	0.13	8	98
AdaBoost	0.61	85	27	40	87	0.16	10	97
Naive Bayes	0.59	38	21	86	36	0.09	5	98

*XGBOOST, eXtremely Gradient Boosting; CatBoost, Categorical Boosting; LightGBM, Light Gradient Boosting; MLP, Multi-Layer Perceptron; SVM, Support Vector Machine; LR, Logistic Regression. KNN, K-Nearest Neighbor; AdaBoost, Adaptive boosting; ACC, accuracy, PPV, positive predictive value; NPV, negative predictive value*.

### Application of the Model

**Figure 3:**
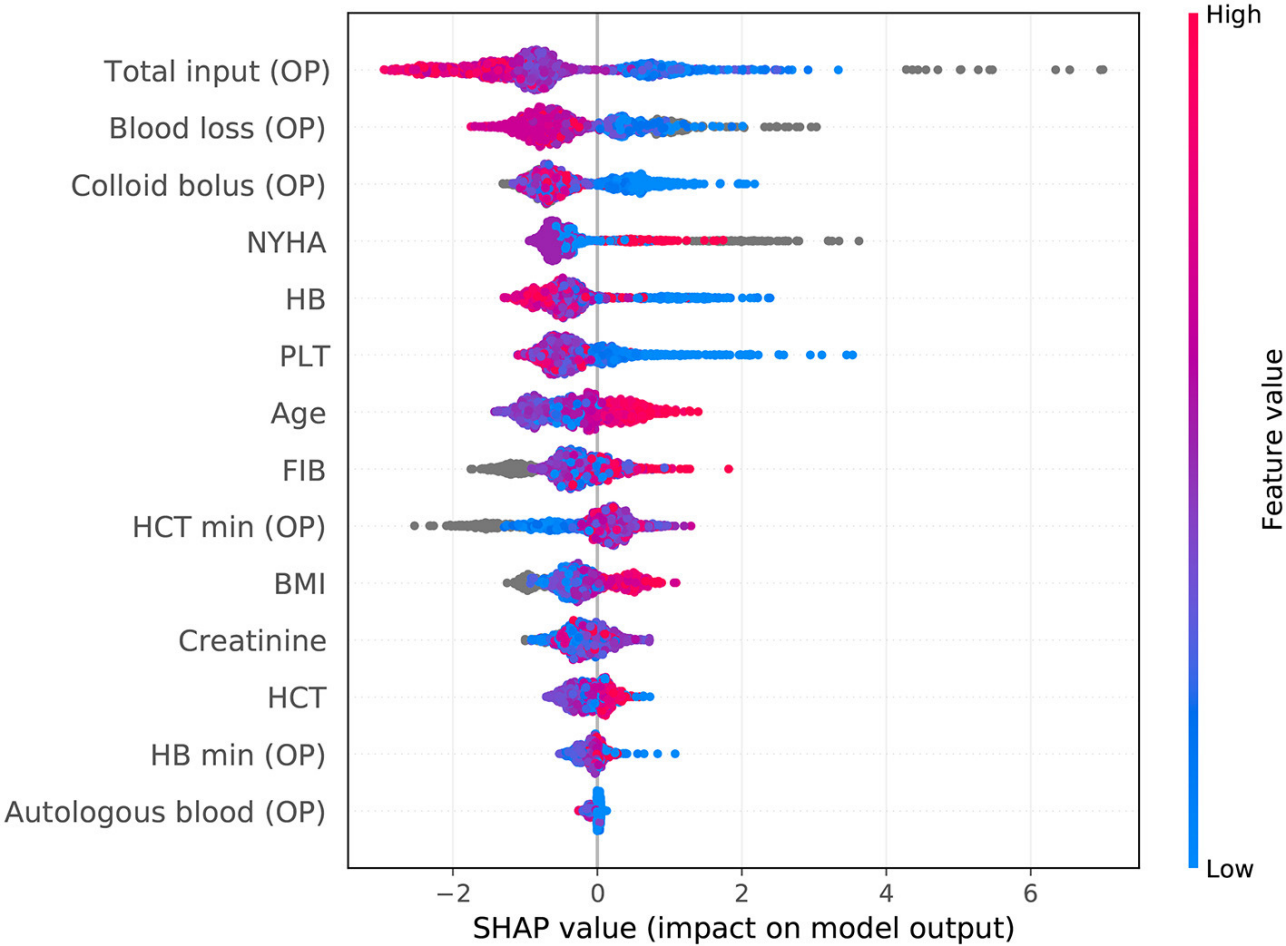
SHAP analysis of the proposed model on the whole cohort. This figure described data from the whole cohort, with each point representing one patient. The color represents the value of the variable; blue represents the smaller value, and red represents the larger value; the horizontal coordinates represent a positive or negative correlation with severe complications risk, with a positive value indicating a good outcome and a negative value indicating a risk of severe complications. The absolute value of the horizontal coordinate indicates the contribution of variables; the greater the absolute value of the horizontal coordinate, the greater the contribution of the variables.

**Figure 4:**
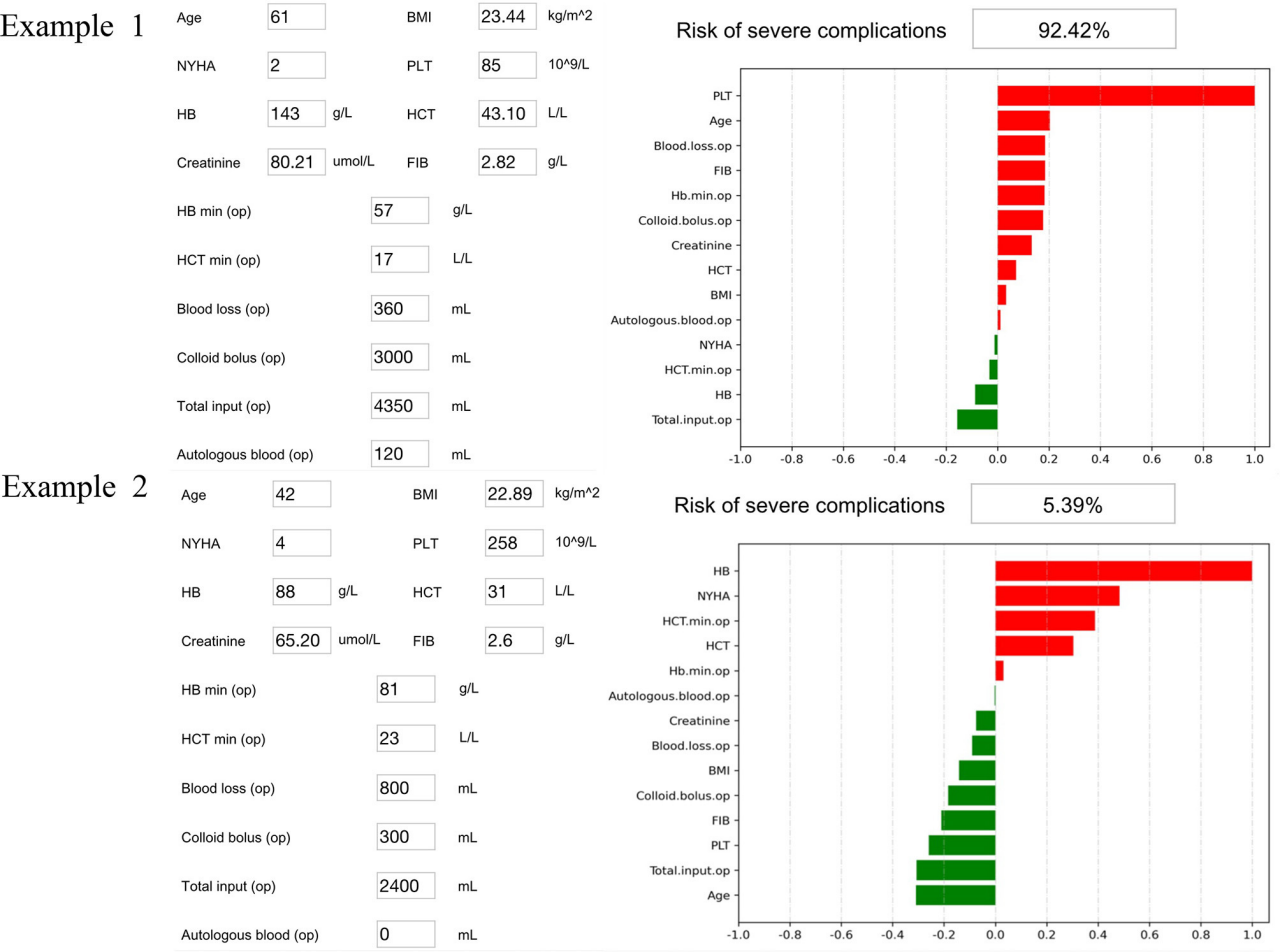
Two examples of website tool usage. Enter the values of 14 key variables to predict the risk of severe complications and show the contribution of each value to the outcome. Example 1 has a higher risk of severe complications, and example 2 may have a better prognosis.

The SHAP package analyzed the entire cohort, and showed the impact of each variable on predicting complications (Figure 3). The preoperative and intraoperation information of a patient was inputted into the model: age 61 years, BMI 23.44 kg/m^2^, NYHA 2, intraoperative blood loss 360 ml, intraoperative colloid infusion 3,000 ml, intraoperative total input 4,350 ml, intraoperative autologous blood collection 120 ml, preoperative Hb 143 g/l, intraoperative minimum Hb 57 g/l, preoperative Hct 43.1%, intraoperative minimum Hct 17%, preoperative PLT 85^*^10^9^/l, preoperative creatinine 80.21, and preoperative FIB 2.82 g/l. The model analyzed that the risk of adverse events in this patient was 92.4%, indicating that the probability of severe complications for the patients was high (Figure 4A, Example 1). The preoperative and intraoperation information of another patient was inputted into the model: age 42 years, BMI 22.89 kg/m^2^, NYHA 4, intraoperative blood loss 800 ml, intraoperative colloid infusion 300 ml, intraoperative total input 2,400 ml, intraoperative autologous blood collection 0 ml, preoperative Hb 88 g/L, intraoperative minimum Hb 81 g/l, preoperative Hct 31%, intraoperative minimum Hct 81%, preoperative PLT 258^*^10^9^/l, preoperative creatinine 65.2 μmoI/l, and preoperative FIB 2.6 g/l. The predicted probability of adverse events in this patient was 5.3%, indicating that the patient had a good outcome (Figure 4B, Example 2). Furthermore, a website was established for clinicians to use the proposed model, http://www.aimedicallab.com/tool/aiml-valvecomp.html. As shown in Supplementary Figure 1, the predicted probabilities are significantly different between the positive and negative groups. If we use 50% as a cut off, our model will achieve a 100% accuracy.

## Discussion

As being mentioned above, the incidence of postoperative complications can be declined with an eligible medical treatment, including a smooth operation, the early prediction of postoperative risks, the provision of appropriate recommendations, and the timely adoption of effective medical measures, which has been explored by many researches (6). In our study, the incidence of mortality in patients with postoperative complications was 7%, and the incidence of mortality in all cardiac surgery patients was 0.4%. The incidence of postoperative complications in all patients was 6.7%.

Based on previous studies, there is a general convergence of the incidence of postoperative complications among different hospitals. Some high-turnover institutions have low mortality rates and may be associated with higher complication rates (17). In other words, surgical patients in high-turnover facilities may experience one or more postoperative complications, but they have a low mortality rate, because these facilities have a higher rate of rescue success (18). Based on the above research studies, the ability to predict, identify, prepare, and implement the management of postoperative risks are vitally important to improve the outcomes of patients. In previous investigations, several kinds of statistic means have been discovered to figure out outcomes, analyze manifestation, and construct models for improving the outcomes of cardiac surgery (19, 20). Researchers who constructed these models were primarily interested in postoperative mortality and rarely predicted other alternative outcomes, such as postoperative complications, so it is really vital to develop an effective measuring system to predict postoperative outcomes. In this study, we introduced machine learning to build the prediction model.

One of the most significant aspect is increasing the area under the receiver operating characteristic (AUROC) curves of predictive models. The AUROC of traditional predictive models is no more than 0.8 or even lower (21, 22). In our research, the model based on machine learning exhibited a perfect performance. Different methods were used to prove that our prediction model has a good predictive effect on several different postoperative complications, all of the AUROCs were more than 0.8, some of which even reached 0.9. This proved that our model has a fantastic predictive effect on postoperative complications.

We also implemented two examples into our predictive model to confirm what variables were important to the predictive model, which can provide guidance for clinicians in making medical decisions, such as how to manage the cardiac surgery. In this study, we identified 14 key indicators that had a significant impact on clinical outcomes, suggesting that clinicians should take care changes in some important variables, such as NYHA, blood loss, and creatinine (23, 24). This research also found that clinicians should pay close attention to changes in blood clotting function and kidney function of cardiac surgery patients. Most importantly, it can indicate to clinicians how likely a patient is to develop complications after cardiac surgery. Based on the above model, we also built an online open website. We can easily obtain the incidence of postoperative complications for a particular patient by entering several important variables in the corresponding column of this website. It is proved that the accuracy of our prediction model is very high, which can provide guidance for clinicians.

Inevitably, our research still leaves some to be desired. On the one hand, this study was a retrospective study with selection bias and confounding factors. We have enhanced the reliability of our results by incorporating multicenter data and performing robust cross-validation. At the same time, we will add prospective studies to our future studies to reduce these errors. A randomized controlled trial associated with this research should be conducted. However, the design of this type of RCT remains unclear. On the other hand, the entire process of machine learning to complete tasks operates in a black box, lacks interpretability, and is not as intuitive and clear as traditional linear models. Our results showed that the machine learning model had incomparable prediction efficiency compared with traditional linear model prediction. This study did not include patients undergoing minimally invasive mitral valve replacement, and this algorithm is not applicable to such patients. Because of the increasing use of this surgical procedure, we will include such patients in subsequent studies. Meanwhile, we have implemented a web page to promote clinical application, which is actually very meaningful and convenient.

In this study, a postoperative complication prediction model after cardiac surgery was exploited based on a machine learning algorithm, with a splendid prediction performance and convenient implementation. This model has the ability to recognize minimal risk of postoperative complications. Meanwhile, the best outcomes of patient prognosis can be achieved through an individualized assessment system. To reduce selection bias, a prospective management database for surgery patients should be built. Based on preoperative and intraoperative variables, machine learning models can be constructed and validated by the variables of surgery patients in the future. Last but not least, to measure the performance of machine learning models, a randomized controlled trial associated with this research should be conducted. It can provide suggestions for clinical work, and reduce the risk of patients and improve patient outcomes.

## Data Availability Statement

The datasets presented in this article are not readily available because. The Ethics Committee did not agree to disclose the data in a public database. Requests to access the datasets should be directed to Leping Liu, 1105380949@qq.com.

## Ethics Statement

The studies involving human participants were reviewed and approved by the Institutional Review Board of the Third Xiangya Hospital of Central South University (NCT03885570). Written informed consent was not required for this study, in accordance with the local legislation and institutional requirements.

## Author Contributions

HJia, YW, HJi, XM, JW, YH, and XW: clinical data collection. HJia and BC: data analysis. RG, HJia, and LL: writing of the article. RG: designing of the study. QZ: construction of the model. All authors contributed to the article and approved the submitted version.

## Funding

This study was supported by the National Natural Science Foundation of China (Nos. 81573091 and 81802668), the Natural Science Foundation of Hunan Province (Nos. 2018JJ3776 and 2017JJ3467), and the Fundamental Research Funds for the Central Universities of Central South University under Grant No. 2020zzts892.

## Conflict of Interest

The authors declare that the research was conducted in the absence of any commercial or financial relationships that could be construed as a potential conflict of interest.

## Publisher's Note

All claims expressed in this article are solely those of the authors and do not necessarily represent those of their affiliated organizations, or those of the publisher, the editors and the reviewers. Any product that may be evaluated in this article, or claim that may be made by its manufacturer, is not guaranteed or endorsed by the publisher.
