# Corrigendum: Machine Learning for the Prediction of Complications in Patients After Mitral Valve Surgery

**DOI:** 10.3389/fcvm.2022.890752

**Published:** 2022-03-24

**Authors:** Haiye Jiang, Leping Liu, Yongjun Wang, Hongwen Ji, Xianjun Ma, Jingyi Wu, Yuanshuai Huang, Xinhua Wang, Rong Gui, Qinyu Zhao, Bingyu Chen

**Affiliations:** ^1^Clinical Laboratory, The Third Xiangya Hospital, Central South University, Changsha, China; ^2^Hunan Engineering Technology Research Center of Optoelectronic Health Detection, Changsha, China; ^3^Department of Transfusion, The Third Xiangya Hospital, Central South University, Changsha, China; ^4^Department of Blood Transfusion, The Second Xiangya Hospital, Central South University, Changsha, China; ^5^Department of Anesthesiology, Fuwai Hospital National Center for Cardiovascular Diseases, Chinese Academy of Medical Sciences, Peking Union Medical College, Beijing, China; ^6^Department of Blood Transfusion, Qilu Hospital of Shandong University, Jinan, China; ^7^Department of Transfusion, Xiamen Cardiovascular Hospital Xiamen University, Xiamen, China; ^8^Department of Transfusion, The Affiliated Hospital of Southwest Medical University, Luzhou, China; ^9^Department of Transfusion, Beijing Aerospace General Hospital, Beijing, China; ^10^College of Engineering & Computer Science, Australian National University, Canberra, ACT, Australia; ^11^Department of Transfusion, Zhejiang Provincial People's Hospital, Hangzhou, China

**Keywords:** machine learning, cardiac valvular surgery, complications, predict, model

In the published article, there were two errors in the affiliations. The first error is that “Haiye Jiang^1†^” should have a new affiliation 2 “Hunan Engineering Technology Research Center of Optoelectronic Health Detection, Changsha, Hunan, China,” and change it to “Haiye Jiang^1, 2†^”. The second error is “Qinyu Zhao^2, 9*^” should have affiliation 2 removed “Department of Transfusion, The Third Xiangya Hospital, Central South University, Changsha, Hunan, China,” and change it to “ Qinyu Zhao^10*^”.

The authors apologize for this error and state that this does not change the scientific conclusions of the article in any way. The original article has been updated.

## Publisher's Note

All claims expressed in this article are solely those of the authors and do not necessarily represent those of their affiliated organizations, or those of the publisher, the editors and the reviewers. Any product that may be evaluated in this article, or claim that may be made by its manufacturer, is not guaranteed or endorsed by the publisher.

