# Supplementary figures and images for: Machine Learning for the Prediction of Complications in Patients After Mitral Valve Surgery
Source: Front Cardiovasc Med. 2021 Dec 16;8:771246. doi: 10.3389/fcvm.2021.771246 (PMC8716451; doi:10.3389/fcvm.2021.771246)

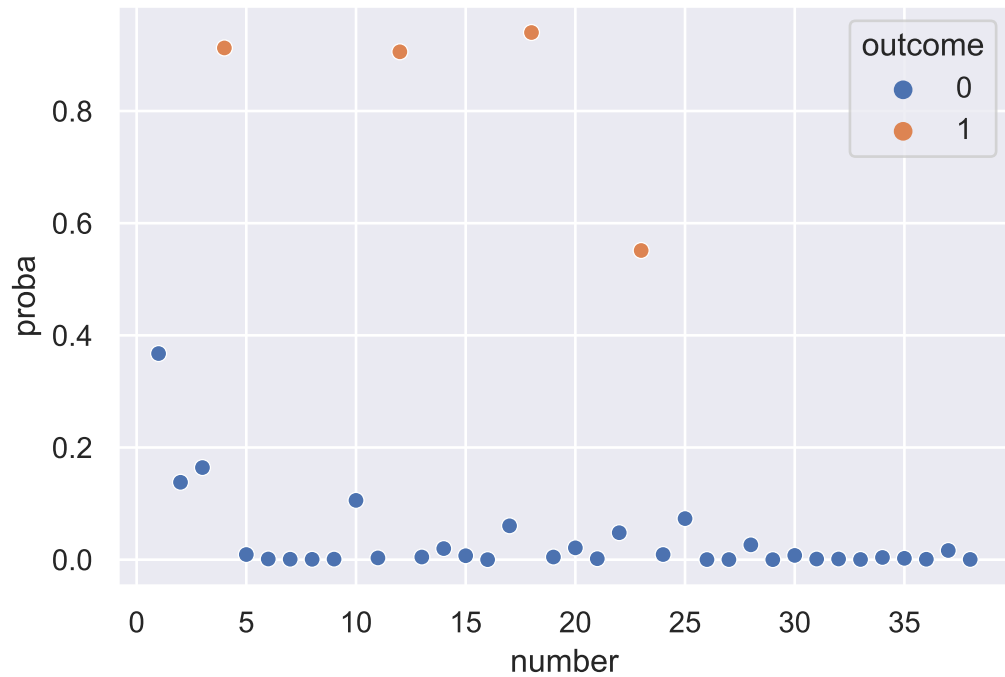

Supplement: Supplementary file 1 [file Image_1.PDF]
